# Adverse Pregnancy Outcomes and Subsequent First-Time Use of Psychiatric Treatment Among Fathers in Denmark

**DOI:** 10.1001/jamanetworkopen.2024.9291

**Published:** 2024-05-01

**Authors:** Frederik Christiansen, Janne Petersen, Ida Holte Thorius, Agnes Ladelund, Espen Jimenez-Solem, Merete Osler, Mikkel Zöllner Ankarfeldt

**Affiliations:** 1 Copenhagen Phase IV Unit, Department of Clinical Pharmacology and Center for Clinical Research and Prevention, Copenhagen University Hospital Bispebjerg and Frederiksberg, Copenhagen, Denmark; 2 Section of Biostatistics, Department of Public Health, University of Copenhagen, Copenhagen, Denmark; 3 Center for Pregnant Women With Diabetes, Department of Endocrinology, Copenhagen University Hospital–Rigshospitalet, and Department of Clinical Medicine, University of Copenhagen, Copenhagen, Denmark; 4 Novo Nordisk A/S, Søborg, Denmark; 5 Center for Clinical Research and Prevention, Bispebjerg and Frederiksberg Hospital, Denmark; 6 Department of Clinical Pharmacology, Copenhagen University Hospital Bispebjerg and Frederiksberg Hospital, Copenhagen, Denmark; 7 Faculty of Health and Medical Sciences, University of Copenhagen, Copenhagen, Denmark; 8 Section of Epidemiology, Department of Public Health, University of Copenhagen, Copenhagen, Denmark

## Abstract

**Importance:**

Becoming a first-time parent is a major life-changing event and can be challenging regardless of the pregnancy outcome. However, little is known how different adverse pregnancy outcomes affect the father’s risk of psychiatric treatment post partum.

**Objective:**

To examine the associations of adverse pregnancy outcomes with first-time psychiatric treatment in first-time fathers.

**Design, Setting, and Participants:**

This nationwide cohort study covered January 1, 2008, to December 31, 2017, with a 1-year follow-up completed December 31, 2018. Data were gathered from Danish, nationwide registers. Participants included first-time fathers with no history of psychiatric treatment. Data were analyzed from August 1, 2022, to February 20, 2024.

**Exposures:**

Adverse pregnancy outcomes including induced abortion, spontaneous abortion, stillbirth, small for gestational age (SGA) and not preterm, preterm with or without SGA, minor congenital malformation, major congenital malformation, and congenital malformation combined with SGA or preterm compared with a full-term healthy offspring.

**Main Outcomes and Measures:**

Prescription of psychotropic drugs, nonpharmacological psychiatric treatment, or having a psychiatric hospital contact up to 1 year after the end of the pregnancy.

**Results:**

Of the 192 455 fathers included (median age, 30.0 [IQR, 27.0-34.0] years), 31.1% experienced an adverse pregnancy outcome. Most of the fathers in the study had a vocational educational level (37.1%). Fathers experiencing a stillbirth had a significantly increased risk of initiating nonpharmacological psychiatric treatment (adjusted hazard ratio [AHR], 23.10 [95% CI, 18.30-29.20]) and treatment with hypnotics (AHR, 9.08 [95% CI, 5.52-14.90]). Moreover, fathers experiencing an early induced abortion (≤12 wk) had an increased risk of initiating treatment with hypnotics (AHR, 1.74 [95% CI, 1.33-2.29]) and anxiolytics (AHR, 1.79 [95% CI, 1.18-2.73]). Additionally, late induced abortion (>12 wk) (AHR, 4.46 [95% CI, 3.13-6.38]) and major congenital malformation (AHR, 1.36 [95% CI, 1.05-1.74]) were associated with increased risk of nonpharmacological treatment. In contrast, fathers having an offspring being born preterm, SGA, or with a minor congenital malformation did not have a significantly increased risk of any of the outcomes.

**Conclusions and Relevance:**

The findings of this Danish cohort study suggest that first-time fathers who experience stillbirths or induced abortions or having an offspring with major congenital malformation had an increased risk of initiating pharmacological or nonpharmacological psychiatric treatment. These findings further suggest a need for increased awareness around the psychological state of fathers following the experience of adverse pregnancy outcomes.

## Key Points

**Question:**

Are adverse pregnancy outcomes associated with incident use of psychiatric treatment among first-time fathers?

**Findings:**

In this Danish register-based cohort study including 192 455 first-time fathers with no history of psychiatric treatment, stillbirth and induced abortion were associated with an increased risk of subsequent initiation of pharmacological or nonpharmacological psychiatric treatment.

**Meaning:**

These findings suggest a need for more awareness of the psychological state of first-time fathers who experience adverse pregnancy outcomes.

## Teaser

This cohort study examines the potential associations of adverse pregnancy outcomes with first-time use of psychiatric treatment among first-time fathers in Denmark.

## Introduction

Becoming a first-time parent is a major, life-changing event and can be challenging regardless of the pregnancy outcome. Research on mothers after giving birth has demonstrated an elevated risk of developing psychiatric disorders, including depression and anxiety, particularly in the context of adverse pregnancy outcomes such as stillbirth or early induced abortion (≤12 wk).^1,2,3,4,5^ These outcomes are associated with risk of initiating psychotropic medication therapy with antidepressants, antipsychotics, and anxiolytics (I.H.T., M.Z.A., K. J. Jensen, PhD, et al; unpublished data; 2024). In recent years, more attention has been paid to men, with several studies suggesting that fathers, like mothers, are at higher risk of developing psychiatric disorders during the pregnancy and postpartum period.^6,7,8,9,10,11,12^ Danish register-based studies have found that for first-time fathers, the prevalence of a hospital admission for a mental disorder was 0.37 per 1000 births at 3 months post partum,^6^ and the cumulative incidence of prescriptions for psychotropic medications was 14.5 per 1000 births during the first year of fatherhood.^6,13^

The factors contributing to the heightened risk of paternal mental disorders in the postpartum period remain largely unexplored, and no studies, to our knowledge, have investigated whether the risk differs in association with adverse pregnancy outcomes. The objective of the present study was to examine the potential associations of adverse pregnancy outcomes with first-time use of psychiatric treatment among first-time fathers. We present both the absolute risks and relative risks, comparing fathers with adverse pregnancy outcomes and those with healthy offspring.

## Methods

This nationwide cohort study follows the incidence of psychiatric treatment among first-time fathers with no history of psychiatric treatment, focusing on those experiencing adverse pregnancy outcomes. The study was approved by the Danish Data Protection Agency. According to Danish law, register-based studies do not require informed patient consent or approval from ethical committees. The study adhered to the Strengthening the Reporting of Observational Studies in Epidemiology (STROBE) reporting guideline.

### Population and Study Design

This Danish nationwide register-based cohort study used data on all registered pregnancies resulting in abortion (spontaneous or induced) or a singleton live-born or stillborn offspring in the period from January 1, 2008, to December 31, 2017. Induced and spontaneous abortions were identified through the Danish National Patient Register^14^ (*International Statistical Classification of Diseases and Related Health Problems, Tenth Revision* [*ICD-10*] codes O04-O06, O021, and O03). A man was identified as the father experiencing an abortion if registered as cohabitant with the pregnant woman in the Danish Population Register. A liveborn or stillborn offspring was identified through the Danish Medical Birth Registry (DMBR), where the fathers are registered as well.^15,16^ For the present study, we included all fathers experiencing either their first fatherhood or an abortion, focusing on the event that occurred first. The date of delivery or the abortion date is referred to as the index date. Follow-up was completed on December 31, 2018.

We excluded fathers with any migration based on a 5-year look-back period before the index date. This exclusion ensured that information about the father’s health care records was available 5 years prior to the index date. To ensure an overall psychiatric healthy cohort based on the 5-year look-back period, we excluded fathers with prior redeemed prescriptions for psychotropic medication registered in the Danish National Prescription Register.^14^ Additionally, fathers with mental and behavioral disorders due to psychoactive substance use (*ICD-10* codes F10- F19, excluding F17) and fathers who had visited a private psychiatrist or a private psychologist were excluded based on data from the Danish National Health Service Register.^17^ Last, we excluded fathers with prior contact to a psychiatric hospital, including emergency visits, outpatient visits and hospitalizations. By excluding all fathers with any prior psychiatric contact, we ensured the cohort consisted solely of individuals without a recorded psychiatric diagnosis in a psychiatric hospital during the look-back period.

### Outcomes

We analyzed outcomes within 1 year after the index date. These included (1) receiving nonpharmacological treatment (psychotherapy [procedure code: BRSP] or private psychiatrist or psychologist visits in the National Health Insurance Service Registry); (2) having a psychiatric hospital contact; (3) redemption of antidepressants (Anatomical Therapeutic Chemical Classification [ATC] codes N06A and N05AN01); (4) redemption of antipsychotics (ATC code N05A, excluding N05AN); (5) redemption of anxiolytics (ATC codes N05B and N05CD); and (6) redemption of hypnotics (ATC codes N05CH01 and N05CF01-03).

### Adverse Pregnancy Outcomes (Exposure Groups)

The reference group was defined as fathers of full-term liveborn offspring not being small for gestational age (SGA) and not having congenital malformations, hereinafter referred to as healthy offspring. The following 9 mutually exclusive pregnancy outcomes define the exposure groups: (1) induced abortion at a gestational age of 12 weeks or less, (2) induced abortion at a gestational age of greater than 12 weeks, (3) spontaneous abortion, (4) stillbirth, (5) preterm birth with and without offspring being SGA, (6) full-term birth with offspring being SGA, (7) offspring with minor congenital malformations, (8) offspring with major congenital malformations, and (9) offspring with congenital malformations and SGA or preterm.

Stillbirth was defined as fetal death after a gestational age of 22 weeks. Information on stillbirth was gathered from the DMBR. Furthermore, spontaneous abortions registered after a gestational age of 22 weeks in the Danish National Patient Register were recoded as stillbirths. Small for gestational age was defined as birth weight below the 10th percentiles stratified by gestational age and sex of offspring. Preterm births were defined as live births before a gestational age of 37 weeks. Information was retrieved from the DMBR. We classified major and minor congenital malformations using the EUROCAT (European Surveillance of Congenital Anomalies) classification system of congenital malformations.^18^

### Covariates

We included baseline characteristics believed to influence adverse pregnancy outcomes and the risk of psychiatric treatment as covariates in the analyses. Data on the age of the father were retrieved from the Danish Civil Registration System.^19^ Information on educational level and income of the father was retrieved from the Danish Education Registers^20^ and the Danish Income Statistics Register,^21^ respectively. Income was divided into quartiles stratified by the year of index date. The highest attained educational level and income were included around the year of the index date, either as educational level attained at the year of the index date or imputed from 1 year before or after the index date in cases of missing information. The educational level was categorized as (1) primary school and high school, (2) vocational school, (3) short- and medium-length further education, and (4) long-length further education. Instead of using further methods like multiple imputation for missing data, our analysis was conducted using only complete cases.

### Statistical Analysis

Data were analyzed from August 1, 2022, to February 20, 2024. The variables are presented as the frequency and percentage for categorical variables and as the median (IQR) for continuous variables. Cumulative incidence plots and unadjusted and adjusted Cox proportional hazards regression models were performed for each of the 6 outcomes, separately, in comparison with the reference group. We adjusted for educational level (grouped), age (continuous), and income quartiles. In the cumulative incidence plots and survival analyses, the fathers were followed from index until the specific outcome, the father died or emigrated, the child died, the mother got pregnant again, or 1 year after the index, whichever came first. We used SAS Enterprise Guide, version 8.3 (SAS Institute Inc), to perform data management and Cox proportional hazards regressions, and R, version 4.3.2 (R Project for Statistical Computing), to create cumulative incidence plots. A standard 2-sided significance level of 5% (*P* < .05) was used.

## Results

### Population Characteristics

Overall, the fathers’ median age was 30 (IQR, 27-34) years. Most of the fathers in the study achieved a vocational educational level (37.1%). Missing values were minimal, affecting only the data on fathers’ age, income, and educational level. The baseline characteristics of the study population, stratified on pregnancy outcome, are presented in Table 1.

**Table 1.** Baseline Characteristics of First-Time Fathers, Stratified by Pregnancy Outcomes

Characteristic	Pregnancy outcome^a^									
	Healthy offspring (n = 132 662)	Induced abortion		Spontaneous abortion (n = 14 190)	Stillbirth (n = 529)	Preterm with or without SGA (n = 8518)	SGA without preterm (n = 16 351)	Malformation		Malformation and SGA or preterm (n = 2844)
		GA ≤12 wk (n = 7409)	GA >12 wk (n = 812)					Minor (n = 3829)	Major (n = 5311)	
Age										
Missing	806 (0.6)	<5	<5	<5	93 (17.6)	42 (0.5)	136 (0.8)	13 (0.3)	31 (0.6)	10 (0.4)
Median (IQR), y	30.0 (27.0-34.0)	26.0 (22.0-33.0)	31.0 (27.0-36.0)	31.0 (27.0-35.0)	30.5 (27.0-35.0)	30.0 (27.0-34.0)	30.0 (27.0-34.0)	30.0 (27.0-34.0)	30.0 (27.0-34.0)	30.0 (27.0-34.0)
Educational level										
Missing	1726 (1.3)	70 (0.9)	7 (0.9)	108 (0.8)	95 (18.0)	104 (1.2)	285 (1.7)	35 (0.9)	72 (1.4)	33 (1.2)
Primary school and high school	33 450 (25.2)	3635 (49.1)	198 (24.4)	3439 (24.2)	152 (28.7)	2129 (25.0)	4428 (27.1)	912 (23.8)	1376 (25.9)	783 (27.5)
Vocational	49 360 (37.2)	2514 (33.9)	297 (36.6)	5370 (37.8)	157 (29.7)	3437 (40.4)	5817 (35.6)	1501 (39.2)	2043 (38.5)	1095 (38.5)
College	29 276 (22.1)	895 (12.1)	193 (23.8)	3185 (22.4)	82 (15.5)	1766 (20.7)	3442 (21.1)	850 (22.2)	1141 (21.5)	556 (19.6)
Graduate	18 850 (14.2)	295 (4.0)	117 (14.4)	2088 (14.7)	43 (8.1)	1082 (12.7)	2379 (14.6)	531 (13.9)	679 (12.8)	377 (13.3)
Income										
Missing	712 (0.5)	5 (0.1)	<5	<5	93 (17.6)	38 (0.4)	120 (0.7)	10 (0.3)	30 (0.6)	8 (0.3)
Quartile 1	29 179 (22.0)	3264 (44.0)	186-190 (23.0)	2931-2935 (21.0)	117 (22.1)	1795 (21.1)	3902 (23.9)	784 (20.5)	1188 (22.4)	658 (23.1)
Quartile 2	35 955 (27.1)	1805 (24.4)	193 (23.8)	3723 (26.2)	107 (20.2)	2374 (27.9)	4332 (26.5)	1026 (26.8)	1488 (28.0)	784 (27.6)
Quartile 3	35 782 (27.0)	1368 (18.5)	229 (28.2)	3870 (27.3)	106 (20.0)	2379 (27.9)	4219 (25.8)	1038 (27.1)	1434 (27.0)	743 (26.1)
Quartile 4	31 034 (23.4)	967 (13.0)	200 (24.6)	3662 (25.8)	106 (20.0)	1932 (22.7)	3778 (23.1)	971 (25.3)	1171 (22.0)	651 (22.9)
Year of pregnancy										
2008-2009	29 697 (22.4)	1765 (23.8)	162 (20.0)	3198 (22.5)	122 (23.1)	1975 (23.2)	3562 (21.8)	633 (16.5)	1063 (20.0)	592 (20.8)
2010-2011	27 266 (20.6)	1604 (21.6)	157 (19.3)	2758 (19.4)	128 (24.2)	1820 (21.4)	3450 (21.1)	745 (19.5)	1093 (20.6)	538 (18.9)
2012-2013	24 954 (18.8)	1415 (19.1)	156 (19.2)	2575 (18.2)	121 (22.9)	1542 (18.1)	3161 (19.3)	757 (19.8)	1096 (20.6)	536 (18.8)
2014-2015	25 494 (19.2)	1422 (19.2)	155 (19.1)	2708 (19.1)	81 (15.3)	1616 (19.0)	3237 (19.8)	914 (23.9)	1178 (22.2)	657 (23.1)
2016-2017	25 251 (19.0)	1203 (16.3)	182 (22.4)	2951 (20.8)	77 (14.6)	1565 (18.4)	2941 (18.0)	780 (20.4)	881 (16.6)	521 (18.3)

^a^ Unless otherwise indicated, data are expressed as No. (%) of fathers. Percentages have been rounded and may not total 100. Due to privacy regulations, cells with fewer than 5 observations are masked.

**Figure 1.**
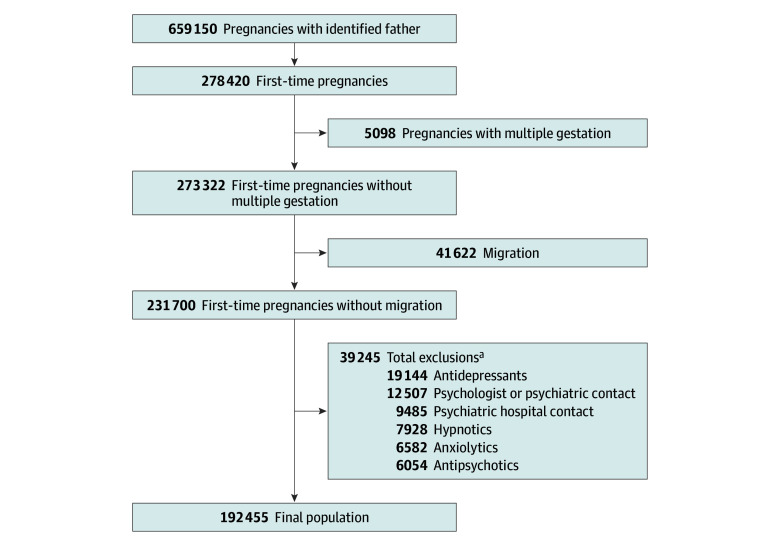
Study Flowchart ^a^Participants could meet more than 1 exclusion criterion.

In total, 273 322 fathers were associated with a first-time abortion or singleton pregnancy from 2008 to 2017. Of these, 41 622 fathers were excluded due to migration within 5 years prior to the pregnancy, and 39 245 were excluded due to a history of antidepressant, antipsychotic, anxiolytic, or hypnotic use, psychiatric hospital contact, or psychologist or psychiatric contact 5 years prior to the index date. The final study population consisted of 192 455 fathers (Figure 1).

Overall, 132 622 pregnancies (68.9%) resulted in healthy offspring, who constitute our reference group. Moreover, 7409 pregnancies (3.9%) of the pregnancies were terminated by induced abortion at a gestational age of 12 weeks or less, 812 (0.4%) were terminated by induced abortion at a gestational age of 13 weeks or more, and 14 190 (7.4%) were terminated by spontaneous abortion. Additionally, 529 (0.3%) of the offspring were stillborn, 8518 (4.4%) were born preterm with or without SGA, and 16 351 (8.5%) were born SGA. Last, 3829 (2.00%) and 5311 (2.8%) of the pregnancies were offspring with a minor and major congenital malformation, respectively, and 2844 (1.5%) resulted in offspring with congenital malformations and SGA or with congenital malformations and born preterm (Table 1).

### Adverse Pregnancy Outcomes and Psychiatric Treatment

For the full cohort, not stratified on exposure group, the 1-year cumulative incidence for receiving nonpharmacological treatment was 0.1%, and the 1-year cumulative incidence of having contact with psychiatric hospitals was 0.4%. The 1-year cumulative incidence of receiving antidepressants was 0.6%; that of receiving hypnotics, 0.4%; that of receiving anxiolytics, 0.2%; and that of receiving antipsychotics, 0.2%.

**Figure 2.**
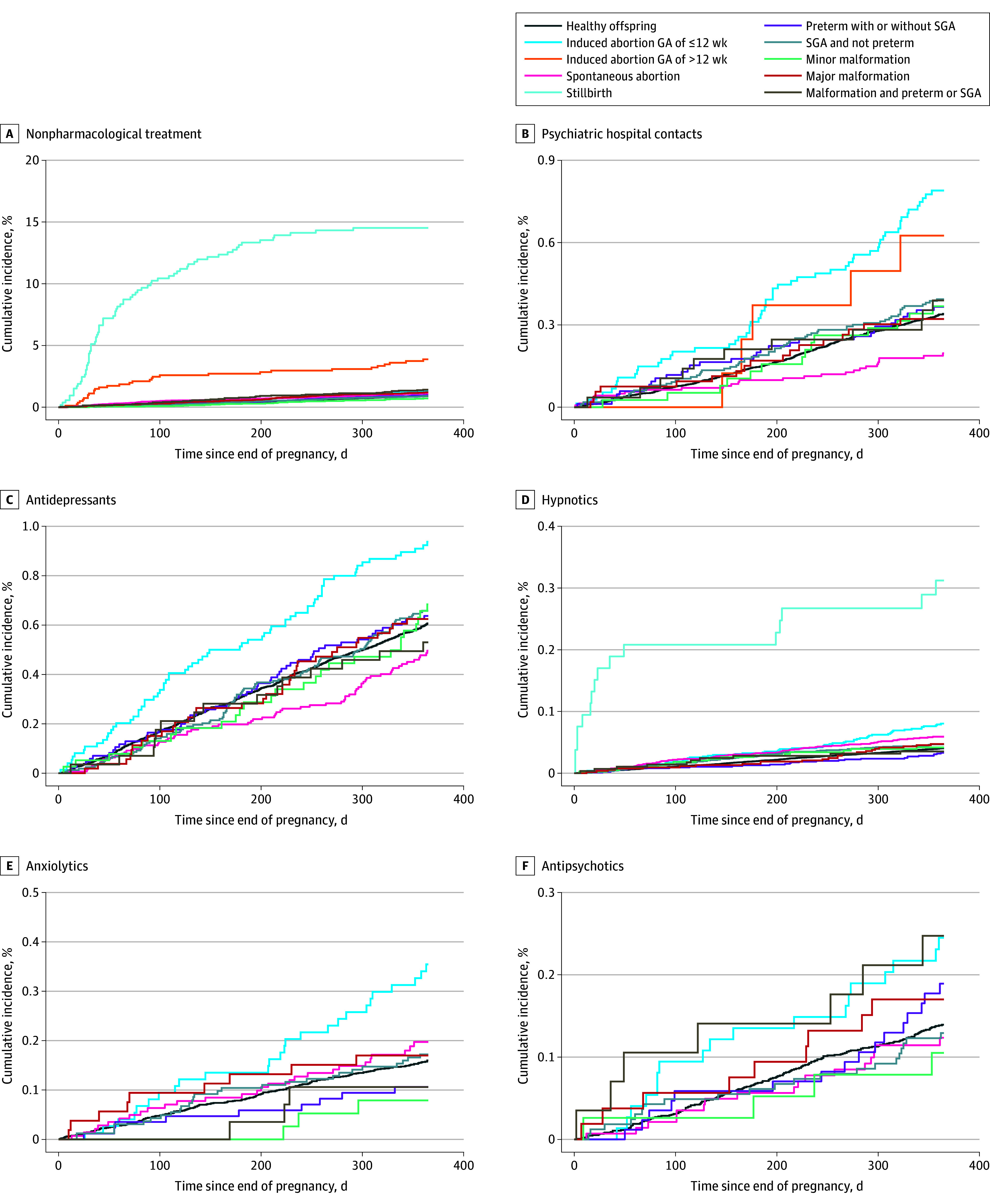
Cumulative Incidence of Psychiatric Treatment, Stratified by Pregnancy Outcome Numbers at risk for each graph are provided in eTable 2 in Supplement 1. Due to privacy regulations, cells with fewer than 5 observations are masked. Anatomical Therapeutic Chemical Classification codes include N06A and N05AN01 for antidepressants; N05A, except N05AN, for antipsychotics; N05B and N05CD for anxiolytics; and N05CH01 and N05CF01-03 for hypnotics. SGA indicates small for gestational age (GA).

Figure 2 and Table 2 show cumulative incidences, number of events, person-times, and adjusted hazard ratios (AHRs) for all exposure-outcome pairs. All AHRs were based on comparison with the reference group of fathers having a healthy offspring. The numbers of fathers at risk in 40-day intervals are shown in eTable 2 in Supplement 1.

**Table 2.** Events, Risk-Time, Cumulative Incidence, and Adjusted Hazard Ratio for Psychiatric Treatment, Stratified for Adverse Pregnancy Outcomes^a^

Pregnancy outcome by treatment^b^	No. of events/person-year	Cumulative incidence, %	AHR (95% CI)
**Nonpharmacological treatment**			
Healthy offspring	1348/129 648	0.90	1 [Reference]
Induced abortion at GA ≤12 wk	94/7233	1.28	1.17 (0.94-1.45)
Induced abortion at GA >12 wk	33/769	3.88	4.46 (3.13-6.38)
Spontaneous abortion	158/13 549	1.10	1.27 (1.07-1.50)
Stillbirth	76/354	14.50	23.10 (18.30-29.20)
Preterm with or without SGA	254/8253	0.97	1.10 (0.88-1.38)
SGA without preterm	199/15 885	0.92	1.02 (0.86-1.21)
Minor malformation	31/3761	0.71	0.81 (0.55-1.18)
Major malformation	118/5159	1.21	1.36 (1.05-1.74)
Malformation and SGA or preterm	175/2719	1.41	1.57 (1.14-2.16)
**Psychiatric hospital contact**			
Healthy offspring	612/129 976	0.34	1 [Reference]
Induced abortion at GA ≤12 wk	59/7252	0.79	1.32 (0.99-1.75)
Induced abortion at GA >12 wk	6/787	0.63	1.49 (0.56-3.98)
Spontaneous abortion	33/13 621	0.20	0.63 (0.42-0.92)
Stillbirth	<5	NA	NA
Preterm with or without SGA	203/8276	0.37	1.10 (0.76-1.58)
SGA without preterm	116/15 922	0.39	1.14 (0.87-1.48)
Minor malformation	18/3767	0.37	1.13 (0.66-1.92)
Major malformation	71/5182	0.32	0.94 (0.58-1.53)
Malformation and SGA or preterm	146/2734	0.39	1.03 (0.55-1.93)
**Treatment with antidepressants**			
Healthy offspring	965/129 782	0.61	1 [Reference]
Induced abortion at GA ≤12 wk	67/7242	0.94	1.09 (0.85-1.41)
Induced abortion at GA >12 wk	<5	NA	NA
Spontaneous abortion	73/13 606	0.50	0.80 (0.62-1.03)
Stillbirth	<5	NA	NA
Preterm with or without SGA	226/8263	0.64	1.06 (0.81-1.40)
SGA without preterm	158/15 904	0.66	1.05 (0.86-1.29)
Minor malformation	30/3762	0.68	1.15 (0.78-1.70)
Major malformation	87/5175	0.62	1.03 (0.72-1.45)
Malformation and SGA or preterm	150/2732	0.53	0.80 (0.47-1.35)
**Treatment with hypnotics**			
Healthy offspring	693/129 922	0.40	1 [Reference]
Induced abortion at GA ≤12 wk	61/7251	0.80	1.74 (1.33-2.29)
Induced abortion at GA >12 wk	<5	NA	NA
Spontaneous abortion	87/13 592	0.59	1.43 (1.13-1.81)
Stillbirth	16/400	3.12	9.08 (5.52-14.90)
Preterm with or without SGA	198/8279	0.33	0.84 (0.57-1.22)
SGA without preterm	126/15 911	0.47	1.15 (0.90-1.46)
Minor malformation	20/3763	0.42	1.06 (0.65-1.75)
Major malformation	79/5180	0.47	1.19 (0.80-1.77)
Malformation and SGA or preterm	146/2733	0.35	0.88 (0.47-1.64)
**Treatment with anxiolytics**			
Healthy offspring	375/130 075	0.16	1 [Reference]
Induced abortion at GA ≤12 wk	27/7267	0.35	1.79 (1.18-2.73)
Induced abortion at GA >12 wk	<5	NA	NA
Spontaneous abortion	32/13 623	0.20	1.17 (0.78-1.76)
Stillbirth	<5	NA	NA
Preterm with or without SGA	181/8287	0.11	0.69 (0.35-1.35)
SGA without preterm	79/15 939	0.17	1.03 (0.69-1.55)
Minor malformation	7/3772	0.08	0.51 (0.16-1.61)
Major malformation	63/5185	0.17	1.09 (0.56-2.13)
Malformation and SGA or preterm	139/2738	0.11	0.67 (0.21-2.08)
**Treatment with antipsychotics**			
Healthy offspring	350/130 094	0.14	1 [Reference]
Induced abortion at GA ≤12 wk	19/7269	0.24	1.07 (0.65-1.77)
Induced abortion at GA >12 wk	<5	NA	NA
Spontaneous abortion	23/13 628	0.12	0.95 (0.58-1.56)
Stillbirth	<5	NA	NA
Preterm with or without SGA	188/8285	0.19	1.41 (0.84-2.35)
SGA without preterm	73/15 943	0.13	0.92 (0.58-1.44)
Minor malformation	8/3771	0.11	0.80 (0.30-2.15)
Major malformation	63/5186	0.17	1.24 (0.64-2.43)
Malformation and SGA or preterm	142/2735	0.25	1.51 (0.67-3.41)

Abbreviations: AHR, adjusted hazard ratio; NA, not applicable; SGA, small for gestational age (GA).

^a^ Adjusted analyses were performed among 189 920 pregnancies. Due to privacy regulation cells with fewer than 5 observations are masked.

^b^ Anatomical Therapeutic Chemical Classification codes include N06A and N05AN01 for antidepressants; N05A, except N05AN, for antipsychotics; N05B and N05CD for anxiolytics; and N05CH01 and N05CF01-03 for hypnotics.

For early and late (>12 wk) induced abortion, the cumulative incidence of nonpharmacological treatment was 1.3% and 3.9%, respectively; the cumulative incidence of psychiatric hospital contact was 0.8% and 0.6%, respectively. For an early induced abortion, the cumulative incidence of antidepressant receipt was 0.9%; the cumulative incidence of anxiolytic receipt was 0.4%. The fathers experiencing an early induced abortion had an increased adjusted risk of receiving treatment with hypnotics (AHR, 1.74 [95% CI, 1.33-2.29]) and anxiolytics (AHR, 1.79 [95% CI, 1.18-2.73]) within 1 year after the abortion, but fathers experiencing a late induced abortion had an increased adjusted risk of receiving nonpharmacological treatment (AHR, 4.46 [95% CI, 3.13-6.38]).

Among fathers of stillborn offspring, the cumulative incidence of receiving nonpharmacological treatment was 14.5%; the cumulative incidence of receiving hypnotics was 3.1%. These fathers also had a significantly increased adjusted risk of receiving nonpharmacological treatment (AHR, 23.10 [95% CI, 18.30-29.20]) and hypnotics (AHR, 9.08 [95% CI, 5.52-14.90]).

For spontaneous abortion, the cumulative incidences for fathers to receive nonpharmacological treatment (1.1%) or medications such as hypnotics (0.6%) or anxiolytics (0.2%) were higher than for fathers of healthy offspring. Furthermore, fathers experiencing a spontaneous abortion had an increased adjusted risk of initiating nonpharmacological treatment (AHR, 1.27 [95% CI, 1.07-1.50]).

Among fathers having offspring with major congenital malformations, the cumulative incidence of receiving anxiolytics and antipsychotics was 0.2%. For fathers having offspring with congenital malformations and being born preterm or SGA, the cumulative incidence of receiving antipsychotics was 0.2%. Having offspring with major congenital malformations had a significantly increased adjusted risk of initiating nonpharmacological treatment (AHR, 1.36 [95% CI, 1.05-1.74]).

Additionally, for fathers having offspring being born preterm with or without an SGA, the cumulative incidence of receiving antipsychotics was 0.2%. There was no increased hazard for fathers having offspring being born SGA, with a cumulative incidence (0.1%) close to the cumulative incidences for a healthy offspring for all outcomes.

eTable 1 in Supplement 1 shows the unadjusted analyses. Although point estimates differed between unadjusted and adjusted analyses, the overall findings were similar, with 1 exception: In the analysis of induced abortions within the first 12 weeks of gestational age, the unadjusted analysis revealed higher point estimates and significantly increased risk compared with the adjusted analysis, likely due to confounding factors in the unadjusted results.

## Discussion

In this Danish nationwide cohort register study of first-time fathers, we compared the risk of fathers initiating first-time nonpharmacological treatment, psychiatric contacts, and treatment with different psychotropic medications within 1 year after experiencing an adverse pregnancy outcome compared with fathers having a healthy liveborn full-term offspring. The results suggest that adverse pregnancy outcomes such as stillbirth and induced abortion were associated with an increased risk of initiation of pharmacological treatment such as hypnotics and anxiolytics and nonpharmacological treatment.

The major finding of this study was a significantly increased adjusted risk of fathers experiencing a stillbirth initiating nonpharmacological treatment and treatment with hypnotics compared with the reference group. The very high AHR of nonpharmacological treatment observed among fathers exposed to stillbirth may point to a greater treatment need among these men. However, it might also reflect that couples who experience stillbirth are more often offered special counseling, which may include psychotherapy. Another major finding was that fathers who experience pregnancies ending in early induced abortions showed an increased adjusted risk of initiating treatments with anxiolytics and hypnotics within a year after the abortion. Last, among fathers experiencing a pregnancy with a late induced abortion or having an offspring with major congenital malformations, there was an increased risk of initiating nonpharmacological treatment.

No previous studies, to our knowledge, have investigated adverse pregnancy outcomes as a determinant for postpartum psychiatric health outcomes among fathers. However, several studies^6,7,8,9,10,11,12^ have assessed the association between the perinatal period and paternal psychiatric episodes. Another Danish register-based study^6^ examining 929 415 births and 543 555 unique fathers found increasing trends of both moderate perinatal psychiatric episodes (identified by psychiatric outpatient treatment in secondary care and by pharmacological treatment in general practice clinics in primary care) and severe perinatal psychiatric episodes (inpatient treatment during the perinatal period). For severe cases, that study demonstrated that a marginally higher proportion of episodes were post partum compared with during the pregnancy (0.10 [95% CI, 0.08-0.11] vs 0.07 [95% CI, 0.04-0.07] per 1000 births, respectively). Additionally, that study did not find any differences between the periods concerning prescriptions for psychotropic medication. Comparing this previous study with our findings is challenging, as we did not compare the pregnancy and postpartum period. Instead, we compare the fathers with and without adverse pregnancy outcomes. Furthermore, our study included expecting fathers who experienced an abortion, and these were not included in the previous study. However, the previous study did seem to include stillbirths, suggesting that our findings concerning stillbirth and major congenital malformations might account for some of the increased trends observed post partum compared with the pregnancy period. On the other hand, many congenital malformations may be detected by the ultrasonographic scans during pregnancy, potentially affecting the father during the pregnancy as well.

A meta-analysis on prevalence of prenatal and postpartum depression in fathers^7^ from 2020 included 47 studies with 20 728 participants. The study found a prevalence of prenatal depression in fathers of 9.8% and a postpartum depression prevalence of 8.8% within 1 year after childbirth. That study underscored the extent of paternal perinatal psychiatric symptoms, highlighting the relevance of further investigation, such as our study. By focusing on the postpartum period, our research aims to uncover some of the mechanisms behind these disorders and symptoms.

In the context of adverse pregnancy outcomes and subsequent psychiatric treatment in fathers, previous studies have been contradictory regarding the timing of birth-related depressive symptoms. With suggestions from no difference between pregnancy and the postpartum period,^11^ an increase in the pregnancy period,^12^ or an increase after the childbirth,^8,9,10^ some of these studies suggest associations between maternal postpartum depression and paternal birth-related depressive symptoms.

In relation to the aforementioned studies^8,9,10,11,12^ investigating the extent and time frame of paternal perinatal psychiatric disorders, our study contributes valuable insights. It suggests that stratifying fathers by pregnancy outcome may be advantageous, and importantly, our findings indicate that even expecting fathers who experience an abortion could be at an increased risk of related psychiatric symptoms, despite never experiencing a childbirth following the pregnancy.

### Strengths and Limitations

A major strength of this study is the large study population from the Danish nationwide registries, which allowed us to analyze 192 455 first-time fathers in our final cohort. The construction of the population allowed us to study incident outcomes, since all fathers had no psychiatric history 5 years before the index date. Additionally, the study only included first-time singleton pregnancies to account for any differences in responses to adverse pregnancy outcomes depending on prior pregnancy experiences. An additional strength is the comprehensive 1-year follow-up period, which enabled us to observe responses beyond the immediate acute stress reactions following the index event.

This study also has some limitations. The absence of detailed context surrounding the pregnancy outcomes may influence interpretation. Induced abortions can elicit mixed emotional responses from parents, ranging from relief to grief, which can significantly influence the subsequent psychiatric reactions. Moreover, some of the included psychotropic medications (eg, hypnotics and antidepressants) can be prescribed for other conditions than psychiatric disorders, such as allergy and pain. Being a register-based study, our analysis was inherently constrained by the scope and detail of the data recorded in the registries. For example, mild symptoms or consultations with a general practitioner not resulting in a prescription or a reference to a specialist were not available. As a result, our study may not have identified fathers experiencing mild psychiatric distress post partum or those with more severe symptoms who did not seek medical help. Therefore, the prevalence of psychiatric symptoms among new fathers is likely underestimated in our findings. Another concern is that some of the identified fathers may have been incorrectly identified as a partner of the associated pregnant woman. Even though this can be the case for some of the identified fathers, the identification method by cohabitation status was the best method available.

Lifestyle factors (eg, smoking and alcohol intake) were not available for consideration; however, by incorporating information about educational level and income, which are associated with lifestyle factors, we were able to account for potential confounders related to lifestyle to some extent. Given the 6 outcomes and 9 exposure categories, there is a risk of observing an association by chance due to multiple testing. No formal adjustment for multiple testing was performed, as the primary aim of this study was to identify potential associations in a field with limited existing research. Future studies need to support the findings of the present study in more detail.

The generalizability of this study was somewhat limited. While we used nationwide data, covering multiple years, the Danish health care system is tax based, with no cost for the patients for both inpatient and outpatient contacts, and there are subsidies for prescription medication and private psychologists. In other health care systems, these costs are typically covered out of pocket or by health insurance. Because of the unique structure of the Danish health care system, it is likely that we were able to identify more adverse health care reactions due to adverse pregnancy outcomes than if we conducted the study in a different setting.

## Conclusions

The findings of this cohort study of first-time fathers in Denmark suggest that fathers experiencing stillbirths or induced abortions or having an offspring with a major congenital malformation had an increased risk of initiating pharmacological and/or nonpharmacological psychiatric treatment. This highlights the necessity of increasing awareness regarding the psychological effects experienced by fathers after adverse pregnancy outcomes and the need for more robust support systems.

## Notes

Supplement 1. **eTable 1.** Unadjusted Hazard Ratio*,* Events Risk-Time, and Cumulative Incidence for Psychiatric Treatment, Stratified for Adverse Pregnancy Outcomes **eTable 2.** Number of Fathers at Risk, Based on 40-Day Intervals

Supplement 2.
Data Sharing Statement
