# Supplementary material for: Adverse Pregnancy Outcomes and Subsequent First-Time Use of Psychiatric Treatment Among Fathers in Denmark
Source: JAMA Netw Open. 2024 May 1;7(5):e249291. doi: 10.1001/jamanetworkopen.2024.9291 (PMC11063801; doi:10.1001/jamanetworkopen.2024.9291)
Supplement: Supplement 1. — eTable 1. Unadjusted Hazard Ratio, Events Risk-Time, and Cumulative Incidence for Psychiatric Treatment, Stratified for Adverse Pregnancy Outcomes eTable 2. Number of Fathers at Risk, Based on 40-Day Intervals [file jamanetwopen-e249291-s001.pdf]

Supplementary Online Content

Christiansen F, Petersen J, Thorius IG, et al. Adverse pregnancy outcomes and subsequent first-time use of psychiatric treatment among fathers in Denmark. *JAMA Netw Open*. 2024;7(5):e249291. doi:10.1001/jamanetworkopen.2024.9291

eTable 1. Unadjusted Hazard Ratio, Events Risk-Time, and Cumulative Incidence for Psychiatric Treatment, Stratified for Adverse Pregnancy Outcomes

eTable 2. Number of Fathers at Risk, Based on 40-Day Intervals

This supplementary material has been provided by the authors to give readers additional information about their work.

© 2024 Christiansen F et al. *JAMA Network Open*.

eTable 1. Unadjusted Hazard Ratio, Events Risk-Time, and Cumulative Incidence for Psychiatric Treatment, Stratified for Adverse Pregnancy Outcomes

Pregnancy outcome	*Events/ Person years*	*Cum. Inc.*	*Un-adjusted HR (95%CI)*	*Events/ Person years*	*Cum. Inc.*	*Un-adjusted HR (95%CI)*
			Non-pharmacological \ treatment			Psychiatric hospital contact
Healthy offspring	1,357/131,353	0.90%		617/131,682	0.34%	
Induced abortion ≤12th week	96/7,301	1.28%	1.42 (1.15-1.76)	60/7,321	0.79%	2.32 (1.77-3.06)
Induced abortion >12th week	33/776	3.88%	4.42 (3.10-6.32)	7/794	0.63%	1.85 (0.77-4.46)
Spontaneous abortion	158/13,653	1.10%	1.23 (1.04-1.46)	33/13,725	0.20%	0.58 (0.39-0.85)
Stillbirth	76/449	14.5%	18.8 (14.9-23.7)	<5 events		
Preterm (with/without SGA)	258/8,354	0.97%	1.09 (0.87-1.36)	207/8,377	0.37%	1.09 (0.75-1.56)
SGA (and not preterm)	199/16,168	0.92%	1.02 (0.86-1.21)	116/16,205	0.39%	1.16 (0.89-1.51)
Minor malformation	31/3,796	0.71%	0.79 (0.54-1.15)	18/3,801	0.37%	1.08 (0.63-1.84)
Major malformation	120/5,230	1.21%	1.35 (1.05-1.74)	73/5,253	0.32%	0.95 (0.58-1.54)
Malformation and (SGA or preterm)	176/2,751	1.41%	1.61 (1.17-2.21)	147/2,767	0.39%	1.17 (0.64-2.12)
			Antidepressants			Hypnotics
Healthy offspring	973/131,487	0.61%		701/131,627	0.40%	
Induced abortion ≤12th week	71/7,308	0.94%	1.55 (1.21-1.98)	61/7,320	0.80%	1.99 (1.52-2.61)
Induced abortion >12th week	<5 events			5/793	0.37%	0.93 (0.30-2.90)
Spontaneous abortion	73/13,710	0.50%	0.80 (0.63-1.03)	88/13,695	0.59%	1.48 (1.17-1.86)
Stillbirth	<5 events			16/495	3.12%	7.99 (4.86-13.1)
Preterm (with/without SGA)	230/8,364	0.64%	1.06 (0.80-1.40)	202/8,380	0.33%	0.83 (0.56-1.21)
SGA (and not preterm)	159/16,186	0.66%	1.09 (0.89-1.33)	126/16,194	0.47%	1.16 (0.91-1.47)
Minor malformation	30/3,797	0.68%	1.12 (0.76-1.66)	20/3,798	0.42%	1.04 (0.63-1.71)
Major malformation	89/5,246	0.62%	1.03 (0.73-1.46)	81/5,251	0.47%	1.18 (0.79-1.76)
Malformation and (SGA or preterm)	151/2,764	0.53%	0.89 (0.53-1.49)	146/2,765	0.35%	0.89 (0.48-1.67)
			Anxiolytics			Antipsychotics
Healthy offspring	381/131,782	0.16%		356/131,800	0.14%	
Induced abortion ≤12th week	28/7,335	0.35%	2.21 (1.47-3.33)	20/7,338	0.24%	1.76 (1.08-2.85)
Induced abortion >12th week	<5 events			<5 events		
Spontaneous abortion	33/13,726	0.20%	1.23 (0.82-1.83)	23/13,731	0.12%	0.89 (0.54-1.46)
Stillbirth	<5 events			<5 events		
Preterm (with/without SGA)	185/8,387	0.11%	0.67 (0.34-1.31)	192/8,386	0.19%	1.37 (0.82-2.28)
SGA (and not preterm)	80/16,221	0.17%	1.08 (0.73-1.60)	73/16,226	0.13%	0.93 (0.59-1.46)
Minor malformation	7/3,806	0.08%	0.49 (0.16-1.54)	8/3,805	0.11%	0.75 (0.28-2.03)
Major malformation	65/5,256	0.17%	1.07 (0.55-2.08)	65/5,257	0.17%	1.23 (0.63-2.39)
Malformation and (SGA or preterm)	139/2,770	0.11%	0.68 (0.22-2.11)	143/2,768	0.25%	1.81 (0.85-3.85)

Unadjusted analyses were carried out in the final population of 192.455 pregnancies.
Due to privacy regulation cells with less than 5 observations are masked.
Antidepressants ATC-codes: N06A, N05AN01. Antipsychotics ATC-code: N05A, except of N05AN. Anxiolytics ATC-code: N05B and N05CD.
Hypnotics ATC-codes: N05CH01, N05CF01-03.
HR: Hazard ratio. SGA: small for gestational age. 95%CI: 95% confidence interval.

© 2024 Christiansen F et al. *JAMA Network Open*.

eTable 2. Number of Fathers at Risk, Based on 40-Day Intervals

Outcome	*exposure*	*0*	*40*	*80*	*120*	*160*	*200*	*240*	*280*	*320*	*360*
Non-pharmacological treatment	Healthy offspring	132,662	132,543	132,394	132,183	131,856	131,485	131,045	130,584	130,117	129,389
Non-pharmacological treatment	Induced abortion ≤12th week	7,409	7,395	7,385	7,368	7,344	7,312	7,279	7,252	7,192	7,077
Non-pharmacological treatment	Induced abortion >12th week	812	798	793	787	786	783	778	775	750	679
Non-pharmacological treatment	Spontaneous abortion	14,190	14,156	14,125	14,092	14,056	14,003	13,932	13,717	12,603	10,870
Non-pharmacological treatment	Stillbirth	529	494	476	463	452	440	435	428	410	371
Non-pharmacological treatment	Preterm (with/without SGA)	8,518	8,442	8,429	8,408	8,385	8,362	8,334	8,301	8,256	8,201
Non-pharmacological treatment	SGA (and not preterm)	16,351	16,325	16,304	16,274	16,230	16,175	16,136	16,074	16,000	15,911
Non-pharmacological treatment	Minor malformation	3,829	3,826	3,825	3,823	3,813	3,804	3,788	3,773	3,758	3,740
Non-pharmacological treatment	Major malformation	5,311	5,293	5,283	5,267	5,250	5,235	5,210	5,183	5,167	5,138
Non-pharmacological treatment	Malformation and (SGA or preterm)	2,844	2,798	2,786	2,773	2,758	2,742	2,736	2,722	2,711	2,689
Psychiatric hospital contact	Healthy offspring	132,662	132,558	132,477	132,355	132,127	131,851	131,500	131,135	130,757	130,111
Psychiatric hospital contact	Induced abortion ≤12th week	7,409	7,401	7,393	7,380	7,364	7,330	7,306	7,286	7,230	7,113
Psychiatric hospital contact	Induced abortion >12th week	812	810	810	808	807	803	799	795	771	700
Psychiatric hospital contact	Spontaneous abortion	14,190	14,181	14,175	14,160	14,133	14,086	14,025	13,819	12,707	10,970
Psychiatric hospital contact	Stillbirth	529	525	525	520	513	504	502	495	476	429
Psychiatric hospital contact	Preterm (with/without SGA)	8,518	8,444	8,433	8,418	8,403	8,383	8,366	8,343	8,304	8,251
Psychiatric hospital contact	SGA (and not preterm)	16,351	16,329	16,309	16,292	16,263	16,219	16,185	16,140	16,076	15,990
Psychiatric hospital contact	Minor malformation	3,829	3,827	3,826	3,825	3,818	3,809	3,794	3,782	3,768	3,752
Psychiatric hospital contact	Major malformation	5,311	5,296	5,291	5,282	5,271	5,258	5,242	5,222	5,207	5,182
Psychiatric hospital contact	Malformation and (SGA or preterm)	2,844	2,801	2,792	2,781	2,771	2,761	2,756	2,747	2,737	2,718
antidepressant	Healthy offspring	132,662	132,521	132,377	132,201	131,932	131,616	131,242	130,853	130,449	129,767
antidepressant	Induced abortion ≤12th week	7,409	7,397	7,385	7,366	7,345	7,321	7,293	7,267	7,212	7,102
antidepressant	Induced abortion >12th week	812	810	810	807	807	804	800	797	773	703
antidepressant	Spontaneous abortion	14,190	14,182	14,170	14,148	14,117	14,070	14,004	13,798	12,679	10,935
antidepressant	Stillbirth	529	525	525	519	513	504	502	494	475	428
antidepressant	Preterm (with/without SGA)	8,518	8,441	8,430	8,416	8,394	8,370	8,347	8,319	8,281	8,228
antidepressant	SGA (and not preterm)	16,351	16,327	16,307	16,283	16,251	16,194	16,163	16,112	16,037	15,947
antidepressant	Minor malformation	3,829	3,826	3,823	3,820	3,815	3,804	3,791	3,775	3,762	3,741
antidepressant	Major malformation	5,311	5,298	5,289	5,276	5,263	5,252	5,230	5,210	5,193	5,166
antidepressant	Malformation and (SGA or preterm)	2,844	2,801	2,792	2,780	2,769	2,759	2,752	2,742	2,730	2,714
Hypnotics	Healthy offspring	132,662	132,534	132,435	132,301	132,065	131,782	131,434	131,071	130,685	130,028
Hypnotics	Induced abortion ≤12th week	7,409	7,401	7,394	7,377	7,359	7,335	7,310	7,285	7,225	7,111
Hypnotics	Induced abortion >12th week	812	809	807	805	805	803	799	796	772	702
Hypnotics	Spontaneous abortion	14,190	14,176	14,161	14,137	14,102	14,053	13,981	13,772	12,660	10,927
Hypnotics	Stillbirth	529	516	515	510	506	497	493	486	467	419
Hypnotics	Preterm (with/without SGA)	8,518	8,443	8,434	8,422	8,407	8,390	8,370	8,346	8,311	8,255
Hypnotics	SGA (and not preterm)	16,351	16,326	16,301	16,282	16,246	16,204	16,172	16,125	16,060	15,979
Hypnotics	Minor malformation	3,829	3,826	3,823	3,820	3,812	3,804	3,791	3,777	3,766	3,751
Hypnotics	Major malformation	5,311	5,297	5,290	5,281	5,270	5,257	5,243	5,219	5,201	5,174
Hypnotics	Malformation and (SGA or preterm)	2,844	2,800	2,790	2,780	2,769	2,760	2,755	2,746	2,736	2,719
Anxiolytics	Healthy offspring	132,662	132,570	132,504	132,396	132,197	131,949	131,638	131,310	130,958	130,343
Anxiolytics	Induced abortion ≤12th week	7,409	7,405	7,399	7,387	7,372	7,351	7,325	7,308	7,254	7,144
Anxiolytics	Induced abortion >12th week	812	810	810	807	805	803	798	796	772	702
Anxiolytics	Spontaneous abortion	14,190	14,183	14,177	14,159	14,133	14,086	14,024	13,819	12,710	10,971
Anxiolytics	Stillbirth	529	525	525	520	514	505	503	496	476	429
Anxiolytics	Preterm (with/without SGA)	8,518	8,446	8,438	8,427	8,413	8,396	8,381	8,357	8,322	8,272
Anxiolytics	SGA (and not preterm)	16,351	16,331	16,317	16,301	16,271	16,237	16,210	16,167	16,106	16,026
Anxiolytics	Minor malformation	3,829	3,828	3,827	3,827	3,822	3,815	3,802	3,790	3,777	3,763
Anxiolytics	Major malformation	5,311	5,298	5,290	5,282	5,271	5,260	5,246	5,229	5,214	5,190
Anxiolytics	Malformation and (SGA or preterm)	2,844	2,802	2,794	2,786	2,777	2,767	2,760	2,751	2,741	2,725
Antipsychotics	Healthy offspring	132,662	132,582	132,517	132,415	132,216	131,969	131,656	131,334	130,989	130,367
Antipsychotics	Induced abortion ≤12th week	7,409	7,406	7,400	7,389	7,372	7,351	7,330	7,312	7,260	7,152
Antipsychotics	Induced abortion >12th week	812	810	809	806	806	804	800	798	773	703
Antipsychotics	Spontaneous abortion	14,190	14,186	14,181	14,165	14,138	14,092	14,030	13,826	12,717	10,982
Antipsychotics	Stillbirth	529	526	525	520	514	505	503	496	477	430
Antipsychotics	Preterm (with/without SGA)	8,518	8,447	8,438	8,426	8,412	8,395	8,380	8,356	8,319	8,266
Antipsychotics	SGA (and not preterm)	16,351	16,330	16,315	16,302	16,278	16,243	16,217	16,175	16,114	16,034
Antipsychotics	Minor malformation	3,829	3,827	3,826	3,826	3,821	3,813	3,801	3,789	3,777	3,762
Antipsychotics	Major malformation	5,311	5,298	5,292	5,284	5,273	5,262	5,247	5,230	5,214	5,190
Antipsychotics	Malformation and (SGA or preterm)	2,844	2,800	2,791	2,783	2,773	2,764	2,759	2,749	2,738	2,721

Fathers were followed from index until the specific outcome, the father died or emigrated, the child died, the mother got pregnant again, or one year after the index, whichever came first
