# Supplementary material for: Adverse Pregnancy Outcomes and Subsequent First-Time Use of Psychiatric Treatment Among Fathers in Denmark
Source: JAMA Netw Open. 2024 May 1;7(5):e249291. doi: 10.1001/jamanetworkopen.2024.9291 (PMC11063801; doi:10.1001/jamanetworkopen.2024.9291)
Supplement: Supplement 2. — Data Sharing Statement [file jamanetwopen-e249291-s002.pdf]

Data Sharing Statement

Christiansen. Adverse Pregnancy Outcomes and Subsequent First-Time Use of Psychiatric Treatment Among Fathers in Denmark. *JAMA Netw Open*. Published May 01, 2024. doi:10.1001/jamanetworkopen.2024.9291

Data
Data available: No

Additional Information
Explanation for why data not available: Data from the Danish registers are third party data, meaning that we as researchers do not hold the data, but have obtained data after application at relevant parties. The Danish data can be applied for at Statistics Denmark (https://www.dst.dk/en/TilSalg/Forskningsservice).
